# Prevalence of dementia and its association with central nervous system infections among older persons in northern Uganda: cross-sectional community-based study

**DOI:** 10.1186/s12877-023-04174-9

**Published:** 2023-09-11

**Authors:** Deo Benyumiza, Edward Kumakech, Jastine Gutu, Jude Banihani, Joshua Mandap, Zohray M Talib, Edith K Wakida, Samuel Maling, Celestino Obua

**Affiliations:** 1 Department of Nursing and Midwifery, Faculty of Health Science, Lira University, Lira, Uganda; 2 Office of Health Professional Education Partnership Initiative – Transforming Ugandan Institution’s Training Against HIV/AIDS (HEPI - TUITAH) program, Faculty of Health Science, Lira University, Lira, Uganda; 3 Department of Medicine, California University of Science and Medicine, San Bernadinio, USA; 4 Office of Research Administration, Mbarara University of Science and Technology, P.O. BOX 1014, Mbarara, Uganda; 5 Department of Psychiatry, Faculty of Medicine, Mbarara University of Science and Technology, Mbarara, Uganda; 6 Office of the Vice Chancellor, Mbarara University of Science and Technology, Mbarara, Uganda

**Keywords:** Dementia, Older persons, Central Nervous System infections, Northern Uganda

## Abstract

**Background:**

Dementia is a condition in which there is deterioration in cognitive function beyond what might be expected from the usual consequence of biological aging. Few studies have been conducted on the prevalence of dementia and its association with central nervous system (CNS) infections among older persons in African settings, particularly in Uganda. Therefore, this study assessed the prevalence of dementia and its association with CNS infections among older persons in Lira District, northern Uganda.

**Methods:**

This was a cross-sectional community-based study in Lira district northern Uganda. The study was conducted in March 2022 among 434 older persons aged 50 and above years who were selected by multistage sampling. Data were collected using an interviewer-administered questionnaire supplemented with information from participant’s medical records and a brief community screening instrument for dementia. The instrument classifies dementia into unlikely, probable or possible dementia. Data were entered in duplicate into EpiData version 3.0, then transferred to Statistical Package for Social Sciences (SPSS) version 23 for statistical analysis.

**Results:**

Our study found almost one in four (23%) of the older persons in Lira district northern Uganda were suffering from probable or possible dementia. Our study further found that older persons in Lira district northern Uganda with a positive history of central nervous system infections (CNS) had nearly five times higher odds of having probable or possible dementia compared to their counterparts (cOR: 4.5; 2.76–7.23; p ≤ 0.001). Being in advanced age of 70 + years (aOR: 2.6; 1.6–4.3; p ≤ 0.001), positive history of CNS infection particularly Herpes simplex virus-1 (aOR: 5.4; 1.4–20.5; p = 0.013), and chronic headache (aOR: 1.9; 1.1–3.1; p = 0.019) were independent predictors of probable or possible dementia among participants in this study.

**Conclusion and recommendations:**

Dementia is a common condition among older persons in Lira district northern Uganda with a prevalence of 23% in our study. Older persons with a positive history of CNS infection had higher odd of developing dementia compared to their counterparts. Having advanced age, cerebral malaria, Herpes simplex virus − 1 (HSV-1) infections, and chronic headache were independent predictors for dementia. These results imply that health assessment for the risk of dementia should include screening for history of CNS conditions particularly cerebral malaria, HSV-1 and chronic headache.

## Study background

Alzheimer’s Disease and Related Dementia (ADRD) remains a public health concern and the leading cause of disability and dependency among older persons worldwide [1]. There were more than 58 million people living with dementia in 2020 with nearly 10 million new cases every year [2]. Over 60% of people with dementia live in low- and middle-income countries [2]. As the proportion of older persons in the population is increasing in nearly every country, the number of people living with dementia is expected to rise from 55 million to 82 million by 2030 and 152 million by 2050 [2]. In sub-Sahara Africa (SSA), by 2017, over 2.13 million people were living with dementia and this was projected to double every 20 years, increasing to 3.48 million and 7.62 million by 2030 and 2050 respectively [3, 4]. By 2017, there was a likelihood of almost 90,000 persons aged 60 years and above suffering from Alzheimer’s disease or other types of dementia in Uganda [3]. According to the latest World Health Organization (WHO) data published in 2020, Alzheimer’s and Dementia deaths in Uganda reached 1,286 or 0.62% of the total deaths [5, 6]. The Alzheimer’s and Dementia’s age-adjusted death rate was 19.36 per 100,000 population and Uganda ranks number 60 in the world regarding the burden of dementia [5–7].

Development of dementia was associated with modifiable risk factors such as physical inactivity, unbalanced diets, lifestyle, tobacco and excessive alcohol use, and infections such as streptococcus pneumonia [8, 9]. Aging is a non-modifiable risk factor that has been greatly associated with dementia. However, there were also cases of early onset dementia [8, 9]. Advanced age is the most significant risk factor for Alzheimer’s disease and dementia, with the risk increasing and prevalence rising exponentially in older populations [10, 11]. Genetic factors, including a family history of the disease and specific gene variants like APOE ε4, have been associated with a higher risk of developing Alzheimer’s disease [10]. Studies have found a strong link between cardiovascular health and the risk of Alzheimer’s disease and dementia, with risk factors such as high blood pressure, diabetes, obesity, high cholesterol, and smoking increasing the likelihood of developing these conditions [6, 9, 10, 12, 13]. Lifestyle factors, such as physical inactivity, poor diet, excessive alcohol consumption, smoking, and social isolation, have also been identified as potential risk factors for dementia [6, 14]. On the other hand, higher education levels and engaging in mentally stimulating activities have been associated with a reduced risk of cognitive decline and dementia [6].

Pharmacological agents and non-pharmacological therapies have helped in decreasing the impact of dementia on people living with dementia, their caregivers, and families in the communities [15].

Dementia currently has no known definitive cure and therefore the use of preventive strategies such as engaging in physical and mental activity, eating low cholesterol diet, avoiding smoking, controlling hypertension, controlling diabetes, avoiding obesity, and avoiding alcohol use are essential for good brain health in addition to early identification to reduce the incidence and severity [16].

Despite the availability of the aforementioned preventative options, a quarter of countries worldwide have no national policies in their health systems for offering screening, diagnosis, treatment, and supporting people with dementia and their families [10]. Pharmacological interventions against dementia include the use of Cholinesterase Inhibitors (such as Donepezil, Rivastigmine, and Galantamine) and N-methyl-D-aspartate (NMDA) antagonist (such as Memantine) to improve cognitive impairment in ADRD at all stages [17].

Uganda has an increasing population of older persons who require special attention to reduce the burden of dementia [18]. Despite this trend, there are no guidelines on the management of ADRD in Uganda. Older persons in Uganda and SSA at large are faced with the additional risk for neuroinfections which biologically can interplay with the immune systems and neurological disorders such as dementia [19]. Investigations into the prevalence of dementia among older persons and its association with central nervous system (CNS) infections such as Herpes Simplex Virus-1 (HSV-1), Herpes Simplex Virus-2 (HSV-2), varicella-zoster virus, meningococcal meningitis and HIV would help in understanding the development of dementia. Identifying other factors associated with dementia among older persons is also key to the prevention of severe forms of dementia. However, previous studies were scarce on the association between CNS infections and dementia among older persons in sub-Saharan Africa.

Lira district located in northern Uganda has a high population of older persons who routinely seek healthcare for various ailments [18]. However, screening for dementia is not a routine practice [20] and therefore, older persons miss out on the care for ADRD [21]. The aim of this study was to determine the prevalence of dementia and its association with CNS infections among older persons aged ≥ 50 years in Lira district northern Uganda.

## Methods

### Study design and setting

This was a cross-sectional community-based study [22] that employed quantitative research methods. The study was conducted in 42 villages of Lira District in Northern Uganda. Lira District is located in northern Uganda at a distance of approximately 337 km from Kampala the capital city of Uganda. The health service delivery structure in Lira district is comprised of both public, private for profit and private not profit health structures. The health service delivery structures of Lira district just like any other district in Uganda is hierarchical and patient referral pathway from the lowest level without physical infrastructure known as the Village Health Teams (VHTs) or Health Centre I through Health centre II, Health centre III, Health centre IV (Primary Health Care centre), General hospital or District hospital through to the Regional Referral Hospital (Lira Regional Referral Hospital). The VHTs or the Health Centre I conducts community mobilization and distribution of health commodities and serves the population of a village or cell of about 50 households or 200–250 people [23]. The Health Centre II which has a physical infrastructure for basic outpatient services, preventive services and limited curative services. It serves the population of a parish or ward of about 5,000–10,000 people. The Health Centre III is designed to provide a broader range of healthcare services compared to Health Center IIs. They offer outpatient care, maternity services, laboratory services, minor surgeries, and inpatient care for a limited number of beds. It serves the population of a Sub County or Division of about 20,000–50,000 people. The Health Centre IVs are higher-level facilities that provide comprehensive healthcare services, including inpatient care, surgery, specialized diagnostics, and emergency services. It serves a County or Constituency population of about 100,000- 200,000 people. The General Hospitals or District Hospitals are higher-level facilities that provide a wide range of specialized medical services, including specialized surgeries, advanced diagnostics, inpatient care, emergency services, and specialist consultations. It serves a district population of about 200,000–500,000 people. The Regional Referral Hospitals (in this case the Lira Regional Referral Hospital) are higher-level facilities that provide specialized and advanced healthcare services to a larger population within a specific region. They are equipped with specialized medical departments, advanced diagnostic and treatment capabilities, and a broader range of medical specialists. It serves the population of a group of districts in a given region of about 1–2 million people. Older persons with probable or possible dementia symptoms may seek and initiate healthcare from any of these health service delivery structures and maybe referred up to the Regional Referral Hospital.

### Study population

The study population was older persons aged ≥ 50 years and their adult relatives or caregivers. Older persons who were very ill were excluded because they couldn’t withstand the study procedures. In this study, being very ill was defined as the inability of the older persons and or the caretakers to speak nor hear nor respond to verbal communication due to their whatever unspecified active medical conditions at the time of the study. Given the diversity of medical conditions that may make older persons or their caretakers unable to speak nor hear nor respond to verbal communications, and the random nature of their occurrence in the community, the criteria did not in a biased manner exclude from the study older persons with certain infectious diseases nor bias the study outcomes.

### Sampling

The WHO cluster probability sampling method, which is the most commonly used sampling method for selecting representative samples in surveys or studies conducted in populations where individual-level sampling is not feasible or practical, was employed to select the villages and participants [24]. The WHO cluster probability proportionate to size survey is a single-stage cluster sampling at the level of clusters in this case villages where the older persons reside within Lira district. The WHO cluster probability sampling method involves several steps. Firstly, the target population, in this case, older persons aged 50 years and above residing within Lira district was defined. Secondly, a sampling frame of clusters or primary sampling units (PSUs), consisting of the 10 sub-counties in Lira district was created. Thirdly, 42 clusters, representing villages across sub-counties, were randomly selected out of the 639 villages in Lira district. Fourthly, secondary sampling units (SSUs), which were households with older persons aged 50 years and above, were defined within each selected cluster. Lastly, households with older persons were randomly selected as SSUs.

The 42 villages were selected from the 639 villages in Lira district using the simple fishbowl random sampling method. Each of the 42 villages was visited by the survey team to conduct the WHO cluster probability proportionate to size sampling of the households with the target older persons. The survey team guided by the village political leader (also known as the local council chairpersons in Uganda) and the VHT member of each village, proceeded to the centre of each selected village. At the centre of the village, a pencil was tossed to determine the direction of the team’s walk for selecting the first household with an older person. Subsequently, the team visited the nearest households with older persons until a maximum of 10 older persons were sampled from each village. After sampling from one village, the team moved to the next nearest village and repeated the aforementioned procedure until the desired sample size of 434 participants was achieved.

### Participant recruitment approaches

Older persons were identified by the VHTs which is the lowest community level health structure also known as Health Center I in the Ugandan health service delivery system. They were listed by their respective villages of residence. The VHTs made use of the potential participant’s national identity cards, voter’s cards, driving permits or past medical forms to determine their age and hence eligibility for the study and included them in a register of potential participants by village. To recruit the participants, their residential homes or households were visited by the survey team with the field guidance of the local council chairpersons and the VHT members. At the households, the potential participants were provided dementia-related health education, counseling, study information and an ethically approved token of appreciation equivalent of US$ 1.4 (one point four United States Dollar) only to appreciate their participation in the study.

The VHTs are community health volunteers who maintain registers of all residents including older persons in their respective villages [23]. In addition to providing sampling frame for potential research participants, the register is used for the mobilization of targeted residents for health education, counseling, screening or testing for diseases. The VHTs also use the register for targeted distribution of health communities such as modern contraceptive methods and linkages of patients from the community to health facilities for clinical healthcare.

### Sample size estimation

Sample size for this study was calculated using Kish and Leslie (1965) formula for single group with a proportion outcome variable [25]. The z-score (Z) was 1.96, with the margin of error at 0.05. The prevalence (p) for dementia among older persons aged ≥ 50 years in Lira district was unknown at the time of the study but there was a previous study conducted in southwestern Uganda that found a dementia prevalence of 20% [11] and therefore, we assumed p of 20% or 0.2. The calculated sample size using the Kish and Leslie formula was 246 participants. This sample was adjusted for a design effect of 1.764 (from a cluster size of about 10.3 or 11 and assumed intra-cluster correlation coefficient of 0.082 for the prevalence of dementia within the clusters) to 434 participants. The number of clusters or villages required was calculated by dividing the sample size by the cluster size that was 434/10.3 or 11 which equaled to 42 clusters or villages.

### Data collection tools

Data on the primary outcome (dementia) were collected using the Brief Cognitive Score for Dementia (BCSD) [26] which has been validated and previously used in community-based study on dementia in Uganda [11]. The adaptation of the BCSD tool to the Ugandan cultural and linguistic context during the previous community-based study [11] has improved its measurement accuracy and reduced the risk of misclassification of the participants. Data on the history of CNS infections were collected using a semi-structured CNS infection questionnaire and confirmed by the participant’s medical discharge forms. Medical discharge forms contain items on the patient’s past medical diagnosis among others. The CNS infection questionnaire was developed for the study based on the literature [11, 27]. The CNS infection questionnaire comprised of items that collected data on the demographic characteristics of the participants (age, sex, marital status, level of education, employment status, religious affiliation, and type of occupation), CNS infections [cerebral malaria, herpes simplex virus- 1 (HSV-1), herpes simplex virus- 2 (HSV-2), varicella-zoster virus, meningococcal meningitis, epilepsy, pneumococcal meningitis, polio, and CNS tuberculosis], and other factors likely to be associated with dementia. The CNS infection questionnaire was reviewed by local content experts and adjusted accordingly to assure face and content validity.

The index participant section of the BCSD tool was used to collect data on the thinking and memory functions of the participants. The BCSD has two sections namely the Community Screening Instrument for Dementia (CSID) section to be responded to by the index participant and the Key Informant section to be responded to by the adult relative or caregiver [26]. The CSID section comprised of 9 items that collected data on cognition function (thinking, reasoning and memory).

### Data collection procedure

Data were collected in March 2022. The Research Assistants (RAs) worked with the VHTs to establish the list of households with older persons aged ≥ 50 years who reside in the selected villages. The VHTs also provided field guide for the RAs to visit the selected households. The objectives of the study and the need for older persons to participate in the study were explained to the potential participants and their caregivers. Participants and caregivers who accepted to participate in the study were taken through the consenting process and written informed consent were obtained from each of them. After consenting, the aforementioned data collection tools were administered to collect the data from the participants and the caregivers. The data collection process took about 25 to 40 minutes per participant. Data collection process across the 42 study villages were conducted by three trained medical RAs over a period of 4 weeks.

### Measures of the key variables

The outcome variable (dementia) was measured using the interviewer-administered Brief Cognitive Screening Tool for Dementia (BCSD). Both the Community Screening Instrument for Dementia (CSID) section, responded to by the index participant, and the Key Informant section, responded to by the index participant’s adult relative or caregiver [26], were administered to establish a comprehensive assessment of the individual’s cognitive status. The possible score ranged from 0 to 9. A score of 0–6 indicated possible or probable dementia, while a score of 7–9 indicated unlikely dementia.

The Key Informant section of the BCSD tool comprised 6 items that collected data on decline in mental functioning, changes in ability to think and reason, forgetting, and physical inability. The scores ranged from 0 to 6. A score of 0–4 indicated unlikely dementia, while a score of 5–6 indicated probable or possible dementia. The scores from both the Index Participant and Key Informant sections of the BCSD were entered into the data analysis software as separate ordinal level variables.

To calculate the prevalence of dementia, the scores from the Index Participant section of the instrument were used, as the scores from the Key Informant section of the instrument were often used to further confirm the cognitive abilities and functioning of the Index Participant based on their close family member or caregiver’s observations and interactions with them.

The potential predictor variables, namely history of CNS illness, chronic headache, middle ear infections, physical activity, familial history of dementia, and history of eating fats and oil-rich foods, were all measured using the interviewer-administered questionnaire as binary-level variables, with “yes” coded as 1 and “no” coded as 0. Other potential predictor variables, such as educational level, current and past occupations of the index participant, were measured as multilevel (that is 3 or more levels of data points) categorical variables.

### Data management and analysis

Data collection tools were checked for completeness from the field before departing the field. A data entry screen for data entry was created in EpiData version 3.0. Data was entered in duplicate. Double data entry validation and corrections of entry errors were conducted before exporting of the data to Statistical Package for Social Sciences (SPSS) version 23.0 for statistical analysis. Data were cleaned for out-of-range and missing values before commencing the data analysis. Categorical variables were summarized with frequency counts and percentages. Bivariate analysis (Chi square statistics and Fisher’s Exact Tests) were conducted at 5% significance level and 95% confidence interval for relationships between the outcome variable (dementia), CNS infections and others factors. Independent variables with a *p*-value ≤ 0.2 for their association with dementia at the binary level analysis were included in the multivariate logistic regression model [28]. History of CNS infection was excluded from the multivariate regression model because of its collinearity with both histories of cerebral malaria and HSV-1 infections. Level of education was excluded from the multivariate regression model because some of its categories had less than the recommended 15 cases of dementia. Therefore, the final multivariate logistic regression model to predict probable or possible dementia included age, physical activity, chronic headache, history of HSV-1 infection, and cerebral malaria. The model fitness was checked using the Hosmer-Lemeshow test at *p* > 0.05. The backward conditional logistic regression method was used to determine variables that were independently associated with the outcome variable. The model fitness improved from 70% in stage 1 with all the 5 potential predictors entered in the multivariate regression model to 98% in stage 2 with physical activity backwardly removed from the model.

### Ethical considerations

The research protocol was reviewed and approved by the Gulu University Research Ethics Committee (with the approval number GUREC-2021-185) to which none of the authors was affiliated because the Lira University Research Ethics Committee (LUREC) wasn’t yet accredited to operate at that time of the ethical application for the study. Administrative permissions were also obtained from the office of the Principal Medical Officer of Lira City, the Lira District Health Office and local council chairpersons of the selected 42 villages for the study. Written informed consents were obtained from the participants. Privacy and confidentiality were maintained throughout the study by administrating the data collection process in a private place, use of codes instead of participant’s names, and password protection of electronic dataset. Hard copy questionnaires were kept under lock and key in the corresponding author’s office that was only accessible to the research team.

## Results

### Social-demographic characteristics of study participants

A total 434 older persons in the age range of 50–94 years were enrolled with 0% nonresponse rate. The 100% response rate was achieved through the use of the multiple community engagement approaches including the use of the VHT members to pre-register all the eligible older persons in their respective villages, provision of health education, counseling and ethically approved incentives to both the VHTs and the participants as tokens of appreciation. The distribution of the index participants by their demographic characteristics is shown Table 1.

**Table 1:** Results of the descriptive analysis for the social-demographic characteristics of the index study participants

Variable (n = 434)	Frequency (%)
**Age**	
50–69 years	234 (53.9)
70 years and above	200 (46.1)
**Gender**	
Female	286 (65.9)
Male	148 (34.1)
**Marital status**	
Single	34 (7.9)
Married	247 (56.9)
Cohabiting	1 (0.2)
Divorced	17 (3.9)
Widow	135 (31.1)
**Religious affiliation**	
Catholic	176 (40.6)
Anglican	202 (46.5)
Muslim	8 (1.8)
Pentecostal assemblies of God	48 (11.1)
**Level of education**	
Primary	176 (40.6)
Secondary	44 (10.1)
Tertiary	25 (5.8)
No formal education	189 (43.5)
**Current occupation status**	
Civil servant	41 (9.5)
Business	40 (9.2)
Subsistence farmer	353 (81.3)
**Recent past occupation of the participants**	
Civil servant	52 (12.0)
Business	63 (14.5)
Subsistence farmer	319 (73.5)
**Active in religious matters**	
No	104 (24.0)
Yes	330 (76.0)
**Staying with someone in the house?**	
No	66 (15.2)
Yes	368 (84.8)
**If yes, who is the person** ^*^	
Spouse	188 (51.1)
Child	126 (34.2)
Grand-child	33 (9.0)
Relative	21 (5.7)
**Type of job before reaching 50 years**	
Subsistence farmer	327 (75.3)
Business	51 (11.8)
Social-worker	56 12.9)

legend: % is the percentage.

### Prevalence of dementia among older persons (≥ 50 years) in Lira District, Northern Uganda from the Index Participant’s CSID score

In our study, 100 out of the 434 older persons were found to have probable or possible dementia which translated into a prevalence of 23.0% (95% CI 19.2–27.3). The majority of the older persons with probable or possible dementia were aged ≥ 70 years [61/100 (61.0%)], females [68/100 (68.0%)], and had no formal education [46/100 (46.0%)]. Furthermore, the majority of the older persons with probable or possible dementia were subsistence farmers [77/100 (77.0%)].

### Dementia symptoms among older persons (≥ 50 years) in Lira district Northern Uganda from the perspectives of the caregivers

In our study, caregivers of older persons most frequently reported that the index participants had problems with ‘thinking and reasoning’ [312 (71.9%)], ῾general decline in mental functioning [294 (67.7%)], ῾forgetting where they have put things’ [275 (63.4%)], and ῾remembering what happened the day before [155(35.7%)]. The least number of caregivers reported that the index participants had difficulties dressing [78(18%)].

### Factors associated with dementia among older persons (≥ 50 years) in Lira district, Northern Uganda

On bivariate analysis (Table 2), the variables that were significantly associated with probable or possible dementia were; the age of the participants (p ≤ 0.001), history of chronic headache (p ≤ 0.001) and the positive history of CNS infections (p ≤ 0.001).

**Table 2:** Results of the bivariate analysis for the factors associated with probable orpossible dementia

Factor	Probable or possible dementia	Unlikely dementia	Univariate analysis statistics		
Social-demographics (n = 434)	Frequency (%)	Frequency (%)	Chi-square	df	***p-*** **value**
**Age (years)**			10.870	1	≤0.001*
50–69	39 (16.7)	195 (83.3)			
70 and above	61 (30.5)	139 (69.5)			
**Gender**			2.322	4	**0.613**
Female	68 (23.8)	218 (76.2)			
Male	32 (21.6)	116 (78.4)			
**Marital status**			1.913	4	**0.752**
Single	9 (26.5)	25 (73.5)			
Married	56 (22.7)	191 (77.3)			
Cohabiting	0 (0.0)	1 (100.0)			
Divorced	2 (11.8)	15 (88.2)			
Widow	33 (24.4)	102 (75.6)			
**Level of education**			5.901	3	**0.117**ℷ
Primary	37 (21.0)	139 (79.0)			
Secondary	7 (15.9)	37 (84.1)			
Tertiary	10 (40.0)	15 (60.0)			
No formal education	46 (24.3)	143 (75.7)			
**Past occupation status**			0.889	2	**0.642**
Civil servant	11 (21.2)	41 (78.8)			
Business	12 (19.0)	51 (81.0)			
Peasant	77 (24.1)	242 (75.9)			
**Religious activity**			2.186	1	**0.107**ℷ
No	30 (28.8)	74 (71.2)			
Yes	70 (21.2)	260 (78.8			
**Staying with someone**			0.009	1	**0.801**
No	16 (24.2)	50 (75.8)			
Yes	84 (22.8)	284 (77.2)			
**History of chronic headache**			10.105	1	≤ 0.001*
No	29 (15.4)	159 (84.6)			
Yes	71 (28.9)	175 (71.1)			
**History of recent middle ear infection**			0.513	1	**0.326**
No	97 (23.5)	315 (76.5)			
Yes	3 (14.3)	18 (85.7)			
**Physically active**			2.479	1	**0.090**ℷ
No	38 (28.1)	97 (71.9)			
Yes	62 (20.7)	237 (79.3)			
**Familial history of dementia**			0.020	1	**0.797**
No	60 (23.5)	195 (76.5)			
Yes	40 (22.5)	138 (77.5)			
**History of eating foods rich in fats and oils**			0.078	1	**0.688**
No	70 (23.6)	226 (76.4)			
Yes	30 (21.9)	107 (78.1)			
**History of CNS illness**			38.767	1	≤ 0.001*
No	51 (15.6)	275 (84.4)			
Yes	49 (45.4)	59 (54.6)			

legend: *Significant p-value. ℷ is p ≤ 0.2 cutoff for the inclusion in multivariate regression model. Df is the degree of freedom.

### Specific CNS infections among the older persons (≥ 50 years) in Lira district Northern Uganda

Of the 434 participants in the study, 108 (24.9%) reported a positive history of CNS infections which was later confirmed by their home-kept medical records. The results of a descriptive analysis of the types of CNS infections among the older persons (Table 3) shows that the top 5 CNS infections were HSV-1 (11.8%), Cerebral malaria (8.5%), HIV (5.5%), HSV-2 (1.8%), Varicella-Zoster (1.2%) and Meningococcal meningitis (1.0%).

### Types of CNS infections associated with dementia among older persons (≥ 50 years) in Lira district Northern Uganda

On bivariate analysis (Table 3), the only type of CNS infection statistically associated with probable or possible dementia was Cerebral malaria (p ≤ 0.001).

**Table 3:** Results of the bivariate analysis for the types of CNS infections associated with dementia

Type of CNS Infection	Probable or possible dementia	Unlikely dementia	Chi-square	df	*p-*value
**Cerebral malaria**			23.892	1	≤ 0.001*
No	79(19.9)	318(80.1)			
Yes	21 (56.8)	16 (43.2)			
**HSV-1**			4.141	1	0.027ℷ
No	82 (21.4)	301 (78.6)			
Yes	18 (35.3)	33 (64.7)			
**HSV-2**			-	-	1.000^f^
No	98 (23.0)	328 (77.0)			
Yes	2 (40.0)	6 (75.0)			
**HIV/AIDS**			0.000	1	0.815
No	94 (22.9)	316 (77.1)			
Yes	6 (25.0)	18 (75.0)			
**Meningococcal meningitis**			-	-	0.229^f^
No	98 (22.8)	332 (77.2)			
Yes	2 (50.0)	2 (50.0)			
**Varicella-zoster virus**			-	-	0.326^f^
No	98 (22.8)	331(77.2)			
Yes	2 (40.0)	3 (60.0)			

legend: *Fisher’s Exact Tests is denoted as f; * Significant p-value. ℷ is p ≤ 0.2 cutoff for inclusion in the multivariate regression model. df is the degree of freedom.

### Predictors for dementia among older persons (≥ 50 years) in Lira district, Northern Uganda

The analysis for collinearity between the potential predictor variables, namely age, engagement in physical activity, history of chronic headache, CNS illness, cerebral malaria, and HSV-1, yielded no significant associations. Not even between having positive histories of chronic headache and cerebral malaria. Therefore, these variables were entered into the multivariate binary logistic regression model as additive and independent factors, as the addition of an interaction term was not indicated. The findings from the multivariate logistic regression (Table 4) indicate that the significant independent predictors for probable or possible dementia were the age of the participants (p ≤ 0.001), history of cerebral malaria (p ≤ 0.001), HSV-1 (p = 0.035) and history of chronic headache (p = 0.036) as independent predictors of dementia.

**Table 4:** Results of the multivariate logistic regression analysis for the predictors of dementia

Potential predictors	Probable or possible dementia	Unlikely dementia	cOR; 95% CI	aOR; 95% CI	*P*-value
**Age of participants**					≤ 0.001*
50–69	39 (16.7)	195 (83.3)	**Ref**		
70 and above	61 (30.5)	139 (69.5)	2.41 (1.41–4.11)	2.5 (1.5–4.1)	
**History of HSV-1**					0.035*
No	82 (21.4)	301 (78.6)	**Ref**		
Yes	18 (35.3)	33 (64.7)	2.0 (1.1–3.7)	2.1(1.1–4.1)	
**History of Cerebral malaria**			**Ref**		≤ 0.001*
No Yes	79(19.9) 21 (56.8)	318(80.1) 16 (43.2)	5.28 (2.64–10.60)	5.6 (2.6–12.0)	
**History of Chronic headache**			**Ref**		0.036*
No Yes	29 (15.4) 71 (28.9)	159 (84.6) 175 (71.1)	2.2 (1.4–3.6)	1.73 (1.04–2.87)	
**Physical activity**					**0.314**
No	38 (28.1)	97 (71.9)	**Ref**		
Yes	62 (20.7)	237 (79.3)	0.7 (0.4–1.1)	0.8 (0.5–1.3)	

legend: * Significant p-value. CI is confidence interval. Ref is reference category. cOR is crude odds ratio. aOR is adjusted odds ratio.

## Discussion

Our study found that almost one in four (23.0%) of the older persons in Lira district northern Uganda were suffering from probable or possible dementia (Table 2). This finding was consistent with a previous study conducted among older persons in southwestern Uganda where they found a 20% prevalence of dementia [11]. The similarity in the prevalence of dementia between northern and southwestern regions of Uganda could be due to the similar disease and demographic profiles of the two regions of Uganda. These findings imply that healthcare for older patients in Uganda should include deliberate assessment for symptoms of dementia as a routine care package.

Our study further found that older persons in northern Uganda with a positive history of cerebral malaria were almost 6 times more likely to have probable or possible dementia compared to their counterparts (Table 3). Previous literature was inconclusive on the association between cerebral malaria and dementia among older persons. It was nevertheless consistent with a related prior evidence suggesting an increased risk for dementia-like neurocognitive deficits and behavioral alterations among cerebral malaria survivors in tropical Africa [29]. The association of cerebral malaria with dementia emphasizes the need for intensified malaria prevention efforts including presumptive treatment targeting older persons.

The current study in Lira district northern Uganda further found that older persons with a positive history of HSV-1 were twice more likely to have probable or possible dementia than their counterparts (Table 4). This finding concurs with the results of two previous studies conducted in Asia and Europe [30, 31] where HSV-1 patients were found to be thrice and twice more likely to develop dementia respectively. The consistent association of HSV-1 infection with dementia emphasizes the need for health system strengthening with anti-HSV agents such as Acyclovir, Famiciclovir, Valacyclovir and Peniciclovir to prevent the reactivation of HSV-1 infection in older persons.

In our study in Lira district northern Uganda, older persons with advanced age of ≥ 70 years were almost thrice more likely to have probable or possible dementia compared to their counterparts (Table 4). The association of dementia with advanced age found in our study is consistent with several previous studies conducted in southwestern Uganda and other SSA countries [11, 32, 33] where advanced age was found to be associated with the increased risk for dementia in all the studies. Advanced age has been a well-documented risk factor for dementia but remains unmodifiable factor as aging is a normal physiological process. The consistent association of advanced age (of ≥ 70 years) with dementia across SSA calls for health systems players to expedite the setup of dementia and memory healthcare programs for deliberate dementia screening, presumptive treatment and management of older persons.

In our study in Lira district northern Uganda, older persons with chronic headaches were found to be twice more likely to have probable or possible dementia than their counterparts (Table 4). This finding conforms with several previous studies conducted in Nigeria [34–36] which explored the relationships between migraine and non-migraine headaches and found significant associations between any headache and dementia. Chronic headache is a symptom with multiple differentials including cardiovascular conditions and therefore, the role of chronic headache in dementia, its prevention, and management in older persons warrants further investigations.

### Study limitations

Our study in Lira district northern Uganda did not include laboratory testing of participants to confirm active nor past CNS infections but relied on the participant’s reported history of CNS infections plus cross checking on any available medical discharge forms of the participants. Key informants who were older relatives or caregivers of the index participants were also involved to provide additional information about the trajectory of the CNS illnesses to dementia of the index participants. This study was a cross-sectional study in nature and as such it is unable to provide any evidence towards the temporality of the association between celebral malaria (and other CNS infections) with dementia, thus there is need for longitudinal studies with long-term follow up to ascertain the temporal and directional aspects of the associations between CNS infections and dementia trajectory among the older persons. More so, the use of questionnaire and key informants to collect data on the potential covariates for the development of dementia such as education level and past employment carried the risk of measurement errors. This was however minimized through the triangulation of data sources which in this study were the older persons, their caregivers, and the medical records. Notably, information on the timing of the CNS infections (whether they were early, mid-life or late-life infections), and their severity (whether they were hospitalized infections or home-managed infections) wasn’t reported in this cross-sectional study because of the issues with validity of such data from the retrospective recall approach. The timing of the infections and their severity could be the future direction of researches investigating the associations between CNS infections and the development of dementia.

### Conclusion and recommendations

Dementia is a prevalent condition with almost one in four of the older persons in Lira district northern Uganda were found suffering from the probable or possible dementia. Older persons with a positive history of CNS infections particularly cerebral malaria and HSV-1 had higher odds of dementia compared to their counterparts. Advanced age ≥ 70 years and history of chronic headache were other independent predictors for dementia. Health professionals should pay keen attention to screening older persons for CNS infections particularly celebral malaria, HSV-1, and chronic headache and manage them early to reduce the likelihood of developing dementia. Further research is recommended to characterize the trajectory of CNS infections to dementia particularly the timing and severity of the infections. Studies identifying other non-CNS conditions and exposures associated with dementia amongst older persons in SSA are also desirable.

## Data Availability

The datasets generated and analyzed during the current study are not publicly available because the data collection is a work in progress but data are available from the Corresponding Author upon reasonable request.

## Abbreviations

ADRD: Alzheimer’s Disease and Related Dementia.
AOR: Adjusted Odds Ratio.
BCSD: Brief Cognitive Score for Dementia.
CHEWs: Community Health Extension Workers.
CI: Confidence Interval.
COR: Crude Odds ratio.
CSID: Community Screening Instrument for Dementia.
HIV: Human Immunodeficiency Virus.
HSV-1: Herpes Simplex Virus – 1.
N: Sample size.
NMDA: N-Methyl – D – Aspartate.
p-value: Significance level.
RAs: Research Assistants.
Sn: Serial number.
SPSS: Statistical Package for Social Sciences.
SSA: Sub-Saharan Africa.
VHTs: Village Health Teams.
WHO: World Health Organization.
